# Engineered hypoxia-responding *Escherichia coli* carrying cardiac peptide genes, suppresses tumor growth, angiogenesis and metastasis in vivo

**DOI:** 10.1186/s13036-021-00269-2

**Published:** 2021-08-03

**Authors:** Mitra Samadi, Keivan Majidzadeh-A, Malihe Salehi, Neda Jalili, Zeinab Noorinejad, Marjan Mosayebzadeh, Ahad Muhammadnejad, Azadeh Sharif khatibi, Shima Moradi-Kalbolandi, Leila Farahmand

**Affiliations:** 1 Recombinant Proteins Department, Breast Cancer Research Center, Motamed Cancer Institute, ACECR, Tehran, Iran; 2 Cancer Biology Research Center, Cancer Institute of Iran, Tehran University of Medical Sciences, Tehran, Iran

**Keywords:** Cardiac peptides, Cytokines, MMP9, Angiogenesis, VEGFR2, CD31

## Abstract

Development of engineered non-pathogenic bacteria, capable of expressing anti-cancer proteins under tumor-specific conditions, is an ideal approach for selectively eradicating proliferating cancer cells. Herein, using an engineered hypoxia responding nirB promoter, we developed an engineered *Escherichia coli* BW25133 strain capable of expressing cardiac peptides and GFP signaling protein under hypoxic condition for spatiotemporal targeting of mice mammary tumors. Following determination of the in vitro cytotoxicity profile of the engineered bacteria, selective accumulation of bacteria in tumor microenvironment was studied 48 h after tail vein injection of 10^8^ cfu bacteria in animals. For in vivo evaluation of antitumoral activities, mice with establishment mammary tumors received 3 consecutive intravenous injections of transformed bacteria with 4-day intervals and alterations in expression of tumor growth, invasion and angiogenesis specific biomarkers (Ki-67, VEGFR, CD31and MMP9 respectively), as well as fold changes in concentration of proinflammatory cytokines were examined at the end of the 24-day study period. Intravenously injected bacteria could selectively accumulate in tumor site and temporally express GFP and cardiac peptides in response to hypoxia, enhancing survival rate of tumor bearing mice, suppressing tumor growth rate and expression of MMP-9, VEGFR2, CD31 and Ki67 biomarkers. Applied engineered bacteria could also significantly reduce concentrations of IL-1β, IL-6, GC-SF, IL-12 and TNF-α proinflammatory cytokines while increasing those of IL-10, IL-17A and INF-γ. Overall, administration of hypoxia-responding *E. coli* bacteria, carrying cardiac peptide expression construct could effectively suppress tumor growth, angiogenesis, invasion and metastasis and enhance overall survival of mice bearing mammary tumors.

## Introduction

Despite the efficacy of conventional chemotherapeutic agents in eradicating cancer cells, unacceptable adverse effects of these compounds, resulted from their non-selective toxicity toward normal cells has mostly limited their broad application in clinic and has directed oncologist toward development of novel interventions with selective toxicity toward cancer cells. Pursuing this goal has resulted in development of two new approaches, namely, molecular targeted therapies and immunotherapies. The goal of the first approach is to minimize observed side effects with selective targeting of gene or proteins, demonstrating unique genetic or epigenetic alterations in comparison to normal cells [1–6]. Unfortunately, targeted therapies developed so far, are suffering from several drawbacks [7, 8]. First of all, these modalities have their own spectra of toxicity, including the ones related to the normal functions of the targeted protein [9]. Second, loaded small molecules may not be sufficiently selective toward cancer cells [10, 11]. Third, application of these approaches is usually associated with resistant and relapse, owing to the secondary genetical alterations or selection of a specific group of cancer cells intrinsically resistance to these cells [1, 3, 4]. Finally, most of the tumors do not express currently actionable genetic alterations [12].

On the other side, immunotherapies, including T-cells reactive toward tumor associated antigens and immune checkpoint blockers, are other formats of targeted therapies, which have recently shown to effectively prolong survival rate of patients suffering from advanced metastatic cancers [13, 14]. Nevertheless, low neoantigen burden of most cancer cells and insufficient infiltration of immune cells to the tumor site, have mostly restricted effectiveness of immunotherapies in clinic. Moreover, the dormant cells residing in low infused and hypoxic regions of tumors will become dominant following chemotherapy or radiotherapy which results in clinical relapse and development of resistance of chemotherapeutic agents [15, 16]. Therefore, development of a novel approaches to be selective toward cancer cells and also effective against those residing in hypoxic areas of tumor is highly necessary.

Contrary to the conventional non-selective and highly toxic chemotherapeutic regimens, specific group of anaerobic bacteria including *Salmonella*, *E. coli*, *Clostridium and Bifidobacterium* can preferentially accumulate in tumor microenvironment, especially, necrotic/hypoxic regions, and upon proliferation, induce tumoricidal activity [17]. In Comparison to other therapeutic modalities, the result of bacterial therapy is not affected by the genetic background of tumors. Moreover, the anti-neoplastic effects of bacteria begin from deep hypoxic regions of tumor and continue through potentiation of innate and adaptive anti-tumoral effects. Unfortunately, most of these bacteria are pathogenic for human and rendering them in to non-toxic forms mostly attenuates their toxicity against cancer cells [18]. Consequently, further interventions are required for improving anti-tumoral activity of these bacteria in their non-pathogenic form [19].

One of the routine strategies for achieving this goal is development of genetically modified non-pathogenic bacteria to synthesize cytolytic proteins (e.g. *Staphylococcus aureus* alpha hemolysin toxin) or enzymes with capability of converting non-toxic prodrugs in to cytotoxic ones (e.g. *E. coli* cytosine deaminase, converting 5-Fluorocytosine in to 5-Fluorouracil) under the control of stimuli specific for tumor environment [20]. Considering hypoxic nature of tumor microenvironment, application of specific promoters capable of controlling gene expression in response to hypoxia is a practical approach for developing engineered non-pathogenic bacteria with improved anticancer activity [21]. Recently, Nasr and Eidgahi have successfully developed an engineered nirB promoter which do not possess any responding regions to nitrites and nitrates, and preferentially and selectively induce protein expression under hypoxic condition [22].

Cardiac natriuretic peptides (CNPs) are group of peptide hormones, produced by 3 distinct genes which are stored as prohormones in body. Among them atrial natriuretic peptide (ANP) prohormone is composed of four peptide hormones, named based on their sequence of amino acids beginning from N-terminal, which include long-acting natriuretic peptide (LANP) comprising the first 30 amino acids, vessel dilator (VD) comprising the 31–67 amino acids, kaliuretic peptide comprising 79–98 amino acids and atrial natriuretic peptide (ANP) comprising 99–126 amino acids of the prohormone [23]. Recently, it has been demonstrated that concentrations of CNPs are raised in different types of human carcinomas including invasive squamous cell carcinoma, malignant pericardial effusion, and small cell lung cancer and administration of CNPs at concentrations higher than physiologic ones [24, 25]. In this context, four cardiac peptides were capable of inhibiting up to 97% of cancer cell’s growth in vitro. Moreover, treating human small-cell lung carcinoma (SCLC) nude mouse with these peptides resulted in suppression of tumor growth in more than 80 % of cases. Similar results have also been reported by treating nude mice bearing xerographs of human pancreatic adenocarcinoma with cardiac peptides. Among these peptides, VDL appears to hold the strongest anticancer property, reducing up to 97% of human prostate cancer cells throughout the first 24 h [26–28].

Considering the brief explanations mentioned above, in present study was focused on developing a cancer targeted therapy based on engineered *E. coli* bacterium capable of expressing cardiac peptides under the hypoxic condition of the tumor.

## Material and method

### Ethics statement concerning animal work

All animal experiments were complied with the ARRIVE guidelines and carried out in accordance to the National Institutes of Health guide for the care and use of Laboratory animals (NIH Publications No. 8023, revised 1978). Mice were euthanized by cervical dislocation following completion of tests. The whole experiment and procedures included were approved by the ethics committee of the Iranian academic center for education, culture and research (ACECR).

### Development of plasmid constructs

A polycistronic cassette, expressing KP (60 bp), VDL (111 bp) and LANP (90 bp) genes in tandem under the control of synthetic nirB promoter was designed based on the ANP preprohormone sequence (Genbank accession number: NP_006163). For visualization of bacteria in tumor sites, another polycistronic system expressing green fluorescent protein (GFP) (714 bp) was linked to the previous cassette through a Lambda transcription termination sequence. A ribosomal binding site (RBS) sequence was also included upstream of the GFP and each of the other three cardiac peptide gene sequences. To facilitate excretion of expressed cardiac peptides from bacteria, an immunoglobulin κ chain signal peptide (IgK) sequence was embedded between RBS and each one of the gene’s sequences. In the next step, a terminating codon was located at the end of each gene’s sequence and a Lambda transcription termination sequence (a rho independent transcription termination sequence) was allocated between the two polycistronic expressing systems to separate expression of corresponding mRNAs from each other. Finally, the whole sequence of the construct, was codon optimized with OPTIMIZER® online software (http://genomes.urv.es/OPTIMIZER) and synthesized by Cinnagen Inc. (Cinnagen Co., Tehran, Iran). DNA sequence of the GFP was retrieved from the GFP commercial plasmid pLOX-EWgfp and synthesized by Cinnagen Inc. The synthesized DNA sequence of GFP was fused to the rest of the construct by SOEing-PCR. Finally, flanking sequences of the pET-32 Ek/LIC vector were added to the endings of the construct performing another round of PCR and the final construct was cloned into the vector based on the previously described method [29].

### Transformation of the cardiac peptide/GFP expressing plasmid DNA (pET-CP/GFP)

The *rpoS (Am) rph-1 λ* ^*−*^ *rrnB3 ΔlacZ4787 hsdR514 Δ (araBAD)567 Δ (rhaBAD)568 rph-1 Escherichia coli* strand, BW25133, was grown in Luria-Bertani (LB) media at 37 °C until mid-log phase, and then harvested at 4 °C. After confirmation of correct cloning, pET-CP/GFP constructs were transformed in to *E. coli* BW25133 strain using calcium chloride method. Bacteria were then maintained and selected in LB media applying 50 mg/ml Ampicillin and 10 mg/ml tetracycline. Confirming function of synthetic nirB promoter, bacteria transformed with pET-CP/GFP were grown overnight on LB medium containing 50 mg/ml Ampicillin and 10 mg/ml tetracycline at 37 °C. The anaerobic condition was induced by bubbling filtered helium gas through the medium for 10 min. Plates were then completely sealed in a way to allow no air passage to the medium and incubation continued for 24 h at 37 °C. Fluorescent microscope imaging was performed to detect GFP protein expression by bacteria at the end of the incubation period.

### Toxicity of bacterial secreted cardiac protein on breast cancer cell line

MCF-7 breast cancer cell line was used for evaluating the anti-proliferative effects of expressed cardiac peptides. Briefly, 96 well microtiter plates were inoculated with 200 μL growth media containing a density of 5 × 10^3^ cells and then incubated for 24 h under 5% CO2 and humidified condition. Bacteria bearing cardiac peptide construct and bacteria without construct were grown on LB agar media under hypoxic condition for 48 h and then, the filtrate of bacteria growth media was added to the cells following ultrafiltration (Amicon®, 5 kDa MWCO, Merck, Darmstadt, Germany) and 24 h dialysis against PBS (MWCO = 2000 Da). After a 48 h exposure period, 10 μL of 3-(4,5-dimethylthiazol-2-yl)-2,5-diphenyl tetrazolium bromide (MTT) (Sigma Aldrich) solution with the final concentration of 0.05 mg/mL was added to the media and following 4 h incubation at 37 °C, whole medium was removed and replaced with 200 μL DMSO to dissolve water-insoluble formazan salts. The Absorbance was then read at 570 nm using a 96-well plate spectrophotometer.

### Annexin V-FITC/7-aminoactinomycin D (7-AAD) flow cytometry

Quantification of apoptosis was carried out by flowcytometry. Briefly MCF-7 cells were treated with 48 h growth media of bacteria bearing construct and bacteria without construct as well as PBS for 48 h and then double stained with Annexin V-FITC/ 7-AAD. Fluorescence was then detected with a FACS Calibur flow cytometer (BD Biosciences, USA). Annexin V^+^/7-AAD^−^ cells were determined as early and Annexin V^+^/7-AAD^+^ cells as late apoptotic cells.

### In vivo breast cancer tumor model

In vivo Breast cancer tumor model was developed based on a previously established method by Noori et al. [30]. A female BALB/c mouse bearing spontaneous mouse mammary tumor (SMMT) was obtained from Iranian institute of Pasteur (Tehran, Iran). Previous data indicated that SMMT effectively resembles characteristics of Iranian patient’s invasive ductal carcinoma [31]. SMMT was then carefully separated, dissected into smaller pieces with sizes of less than 0.5 cm^3^ and subsequently, transplanted to 6–8 weak old healthy syngeneic BALB/c mice by surgery. Twelve days after transplantation, when tumors reached to the size of 500 mm^3^, doses of 10^7^ and 10^8^ cfu BW25133 bacteria bearing cardiac peptides and GFP expressing construct, and 10^8^ bacteria without construct per gram body weight of mice were injected systemically to each mouse through the tail vein. Mice in control group received intravenous injections of PBS. A total of three Injections, each with a 4-day interval was performed throughout the study. To evaluate the effect of treatment on suppressing tumor growth, tumor volume was measured on predetermined intervals according to the following formula:

$$ \mathrm{V}\left({\mathrm{mm}}^3\right)={\mathrm{a}}^2\times \mathrm{b}\times 0.5 $$

In which a and b represent the shorter and longer diameters (mm) of the tumor respectively. The results were then compared to the PBS and bacteria without construct receiving groups.

### Cytokine/chemokine profile measurement by multiplex enzyme linked immunosorbent assay (ELISA) array

Alterations in expression of major pro- and anti-inflammatory cytokines and chemokines was evaluated by multiplex ELISA kits (Cat No. MEM-004A, Qiagen) 24 days after injection of engineered bacteria. Briefly, immediately after completion of treatment period, peripheral blood of mice treated with either of PBS or 10^8^ cfu bacteria bearing construct were collected in endotoxin-free EDTA containing collection tubes (BD Vacutainer Plus, US). Plasma was then collected by centrifugation of blood samples at 2500×g for 10 min and kept at − 80 °C until further use. For analysis, 50 μL of thawed samples was added to each well of their respective row. The ELISA procedure was continued according to the manufacturer’s instructions and at the end, absorbance of the 96-well plate was read at 450 nm and 570 nm utilizing a plate reader. For correction of auto-fluorescence, the 570 nm absorbance was subtracted from final absorbance readings. Expression of each cytokine and chemokine was reported as the fold increase above PBS receiving group levels.

### Histological observations and biodistribution of bacteria in normal and tumor tissues

Biodistribution of bacteria in tumor site and normal tissues was carried out 48 h after tail vein injection of 10^8^ cfu BW25133 bacteria per gram body weight of mice. Liver, as one of the most abundantly perfused organs of the body with blood, and tumors were first excised and fixed in 10% formalin solution. The specimens were then paraffin embedded, sectioned and stained with gram stain and visualized under × 100 magnifying lens, using a light microscope. For counting the number of bacteria in normal and tumor tissues, samples were carefully weighed, homogenized in PBS and cultured on LB agar. Bacterial concentration was determined by counting the number of CFUs after 24- to 48 h incubation of plates at 37 °C. Pathological sections were also obtained from specimens and stained with hematoxylin and eosin (H&E) solution to evaluate cytotoxic damage induced by cardiac peptides. Presence of *E. coli BW25133* in sections was also confirmed by visualization of GFP under fluorescent microscope.

### In vivo fluorescence reflectance imaging

Fluorescence reflectance imaging was utilized to initially evaluate whether bacteria could specifically localize in tumor sites and then to monitor time-course of GFP expression as the indicator of successful expression of cardiac peptides in tumor site under hypoxic condition. Briefly, after intravenous injection of PBS (control group) or a single dose of 10^8^ cfu/g engineered *E. coli* BW25133 strain to the mammary tumor bearing mice, In vivo live fluorescence reflectance imaging was performed on days 0, 1, 3 and 6 post injection using the FlouVison fluorescence planar imaging system (Tajhizafarinan Noori Parseh Co., Tehran, Iran). Images from whole body were taken by placing anesthetized mice in the supine position, in the center of the imaging cassette, inside the scanning field of the system. After proper positioning of animals, the imaging cassette was set to the appropriate depth to finely confine anesthetized mice. Finally, the animal body was subjected to the laser beam and emitted non-uniform fluorescent beams were recorded using a highly sensitive thermoelectrically cooled CCD camera located on the same side of the animal.

### IHC and histological examination

At the end of the 24-day treatment period, mice receiving PBS or 10^8^ cfu/g of body weight engineered bacteria were sacrificed and tumors with equivalent dimentions from both groups were selected for further immunohistochemical analyses. Collected tumors were cut in to 7 μm thick sections on a microtome and stained using anti-Ki67 monoclonal antibody (ab15580, Abcam, USA), a marker for tumor proliferation; anti-MMP9 monoclonal antibody (ab38898, Abcam, USA), an enzyme involved in inducing angiogenesis and metastasis; anti-VEGF receptor 2 monoclonal antibody (ab2349, Abcam, USA), a proangiogenic growth factor; anti-CD31/PECAM-1 monoclonal antibody (ab24590, Abcam, USA), recognizing platelet–endothelial cell adhesion molecule-1 (PECAM-1) expressed on surface of endothelial cells; anti-CD8 monoclonal antibody (ab209775, Abcam, USA), an specific marker for cytotoxic T-cells; anti-CD4 monoclonal antibody (ab221775, Abcam, USA), an specific marker for helper T-cells; and their specific horseradish peroxidase-conjugated secondary antibodies. Antigen recovery was performed in citrate buffer pH 6.0 and then blockade of endogenous peroxidase, as well as non-specific proteins was performed by 40 min incubation in 3% of H_2_O_2_ and another 40 min in 3% FBS. At the end, tumor sections were incubated with mentioned antibodies and extend of antigen expression was evaluated using horseradish peroxidase (HRP)-conjugated streptavidin and 2-Solution DAB kit (Life Technologies) according to the companies’ manual instructions. Finally, each antigen’s expression level was quantified using ImageJ software (NIH, Bethesda, MD, USA). Results were expressed as the mean of at least 5 tumor sections for each antigen.

H&E staining was also performed for evaluating tumor infiltrating lymphocytes (TILs) according to the previously established protocols. Initially, percentage of stromal lymphocytes were determined by two separate observers and then stromal TILs were measured as the percentage of immune cells with mononuclear immunological infiltrate characteristics. Findings were classified based on three cut-off points for TIL proportions including 10, 30 and 50%.

### Statistical analysis

Data are represented as mean ± standard deviation. Hypothesis testing was carried out applying Student’s *t*-test and results were considered to be significant when *P* < 0.05. Survival analysis was done by drawing Kaplan–Meier curves and comparing groups with log-rank test. Hazard ratios were calculated by performing Cox’s proportional hazards analysis.

## Results

### Design of construct and expression of cardiac peptides and GFP

**Fig. 1:**
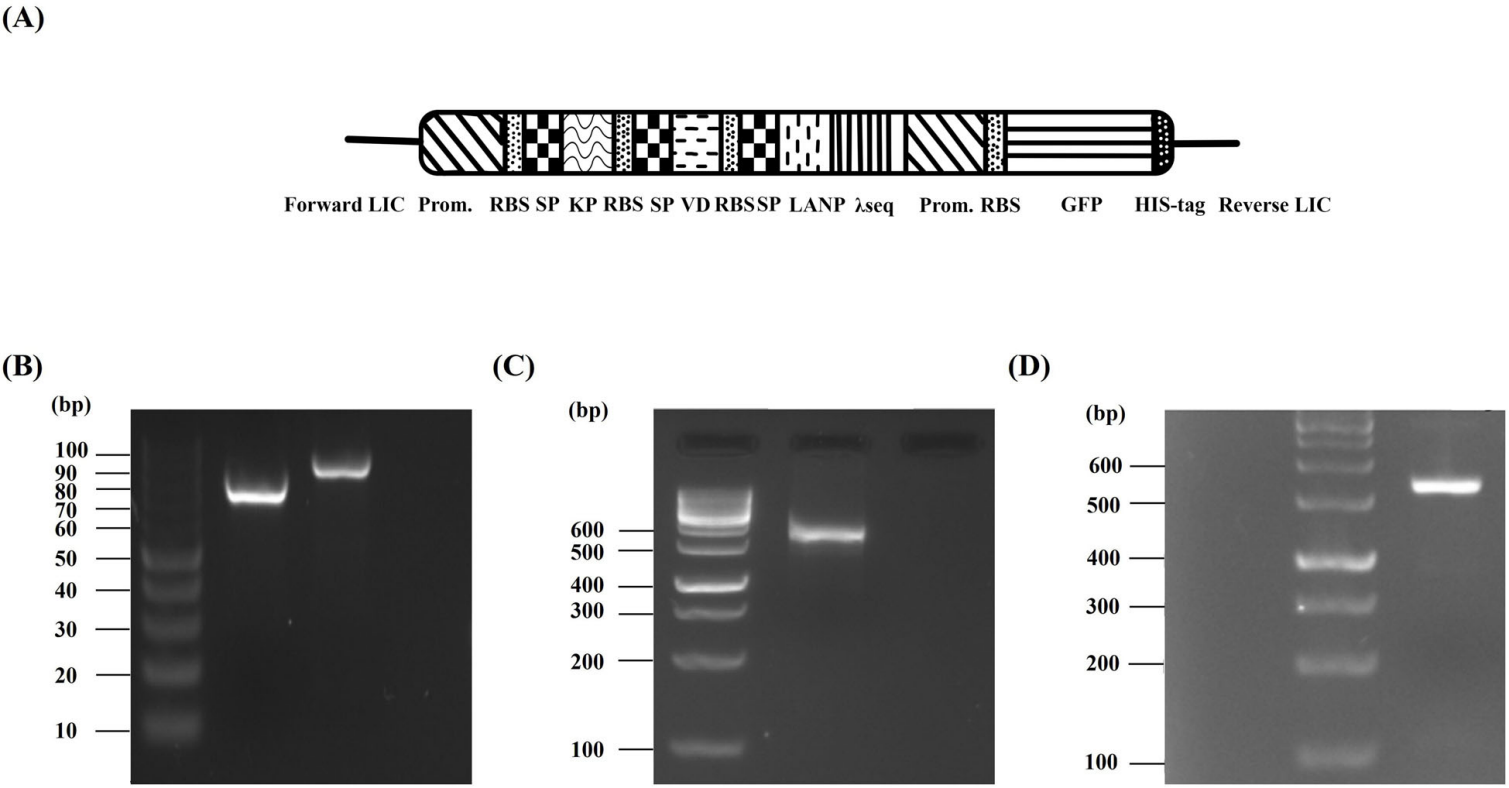
(**A**) The overall structure of the construct co-expressing the cardiac peptides and GFP under the control of synthetic nirB promoter and primers for three PCR. (**B**) results of GFP and Cardiac peptide PCR. Lane 1: the DNA size marker, Lane 2: result of GFP PCR, Lane 3: result of cardiac peptides PCR, Lane 4: Negative control (**C**) Result of SOEing PCR. Presence of a single band at 1500 bp in electrophoresis confirmed correct performance of SOEing PCR. Lane 1: the DNA size marker, Lane 2: result of SOEing PCR Lane 3: Negative control (**D**) Results of colony PCR, confirming the presence of constructs in recombinant bacteria. Lane 1: The negative control (prepared PCR mixture applying water instead of DNA template), Lane 2: the DNA size marker, Lane 3: the positive colonies comprising the cardiac hormone expressing construct

**Fig. 2:**
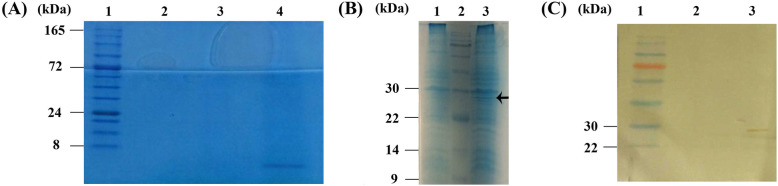
Results of SDS-PAGE analysis. **A** Presence of a single band at about 5 kDa confirmed expression of cardiac peptides. Lane 1: protein marker, Lane 2: before induction of hypoxic condition, Lane 4: 24 h after induction of hypoxic condition. **B** The single band at 27 kDa relates to the molecular weight of GFP which is absent in the bacterial samples prior to induction of hypoxic condition. Lane 1: before induction of hypoxic condition, Lane 2: protein marker, Lane 3: 24 h after induction of hypoxic condition. **C** results of western blot analysis confirming the correct expression of GFP protein. Lane 1: protein marker, Lane 2: before induction of hypoxic condition, Lane 3: 24 h after induction of hypoxic condition

The schematic structure of the construct co-expressing the cardiac peptides and GFP under the control of synthetic nirB promoter has been provided in Fig. 1A. Amplification of cardiac peptides and GFP polycistronic cassettes was performed by specific sets of primers provided in Table 1 and confirmed by presence of two sharp lanes at 750 bp and 850 bp in electrophoresis gels (Fig. 1B). Performing SOEing PCR with new sets of primers, the PCR products of previous step were unified in a new cassette and confirmed by spotting a single band at 1500 bp in electrophoresis gels (Fig. 1C). Results of colony PCR also confirmed the presence of construct in recombinant bacteria (Fig. 1D). Following induction of anaerobic condition, collected supernatant of bacteria was concentrated by ultrafiltration (MWCO = 5 kDa) and the filtrate was dialyzed for 24 h against PBS and then subjected to SDS-PAGE analysis to evaluate expression of cardiac peptides. As demonstrated in Fig. 2A, presence of a single band at about 5 kDa confirmed expression of cardiac peptides. Owing to the very close molecular weight of the cardiac peptides (KP = 2.1 kDa, VDL = 3.93 kDa and LANP = 3.4 kDa), three single bands could have been overlapped together and it is not surprising to observe only one band in SDS-PAGE analysis. Extracted proteins from cytoplasmic compartment of bacterial pellets were also subjected to SDS-PAGE analysis for evaluation of expression of GFP. The band at 27 kDa relates to the molecular weight of GFP which is absent in the bacterial samples non-transformed with the cassette (Fig. 2B).

**Table 1:** Sets of primer used for amplification of cardiac peptides and GFP constructs, as well as the ones used for SOEing PCR

Sets of primer	Sequences (5′ → 3′)
**Cardiac peptides amplification**	**Forward:** GACGACGACAAGATGGGCGAATTGAAGCTGCCCTT **Reverse:** TGATTACGCCAAGCTGCCCTT
**GFP amplification**	**Forward:** AAGGGCAGCTTGGCGTAATCAAAGGAGATATACATATGGTGAGCAAGGGCGAGG **Reverse:** GAGGAGAAGCCCGGTTAGTGGTGGTGGTGGTGGTGCTTGTACAGCTCGTCCATGCCG
**SOEing PCR**	**Forward:** GACGACGACAAGATGGGC **Reverse:** GAGGAGAAGCCCGGTTAGTG

### Anti-proliferative and pro-apoptotic effects of bacteria secreted cardiac peptides

**Fig. 3:**
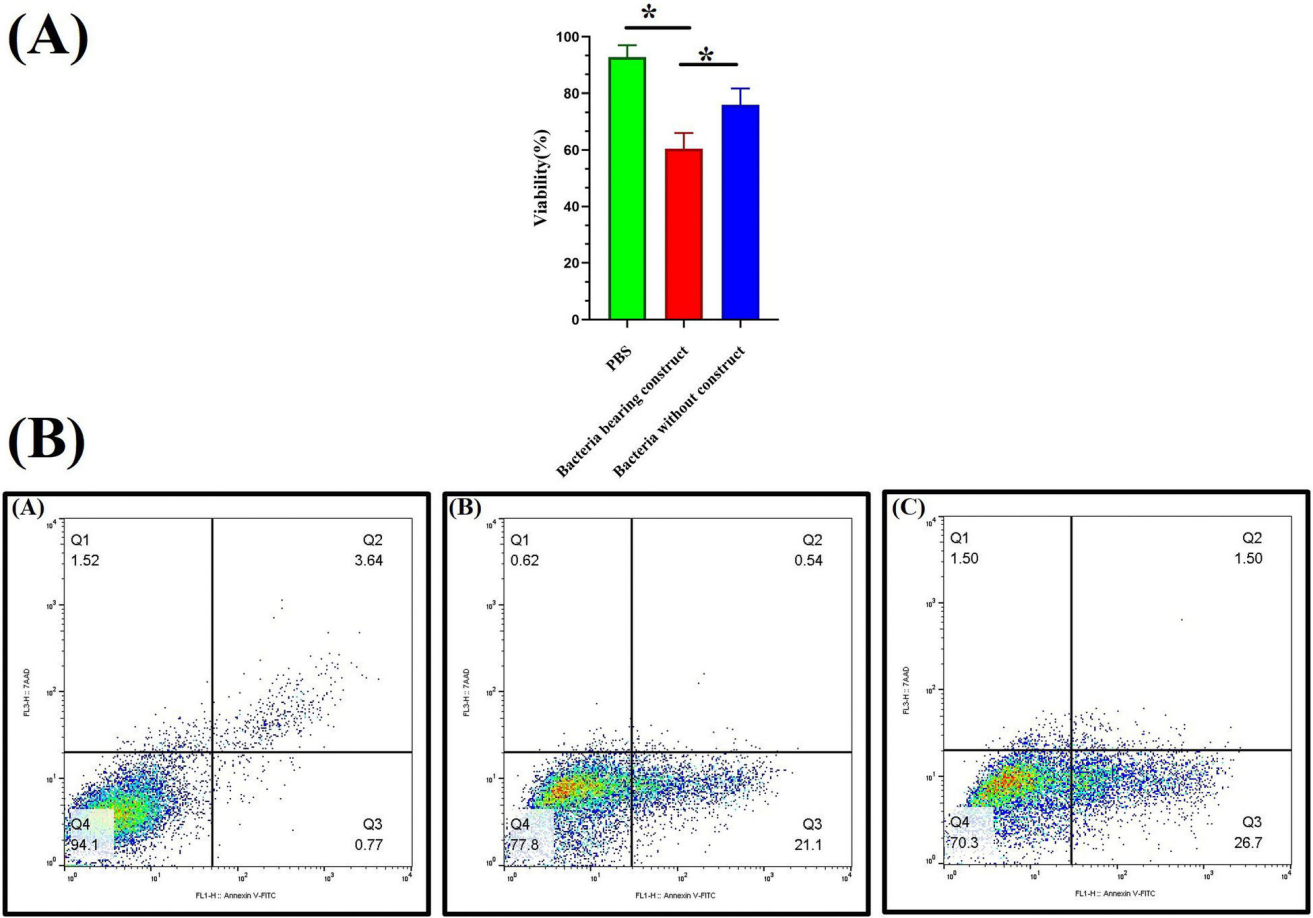
**A** In vitro cytotoxicity of purified cardiac peptides from 48 h growing media of bacteria bearing construct under hypoxic condition. **B** The results of Annexin-V/7-AAD flow cytometry analysis. Treating MCF-7 cells with cardiac peptides mixture significantly increased rate of apoptosis compare to PBS receiving group (Left: PBS, Middle: media of bacteria without construct, Right: cardiac peptides purified from media of bacteria bearing construct)

Incubation of MCF-7 human mammary carcinoma cells with ultra-filtrated and dialyzed growth media of bacteria bearing construct under hypoxic condition for a 48-h period, promoted both cell death and apoptosis. The results of MTT assay demonstrated that administration of the concentrated and purified cardiac peptides from engineered bacteria growing medium could reduce proliferation rate of MCF-7 cells to about 60% of the non-treated control group which was significantly higher than those observed with growth media of bacteria without construct (about 25% inhibition in growth rate; Fig. 3A). The Annexin-V/7-AAD flow cytometry analysis also demonstrated that treating MCF-7 cells with the growth media of bacteria bearing construct could moderately increase rate of apoptosis compare to the growth media of bacteria without construct and PBS treated group (Fig. 3B).

### Preferential colonization of *E. coli BW25113* within tumor

**Fig. 4:**
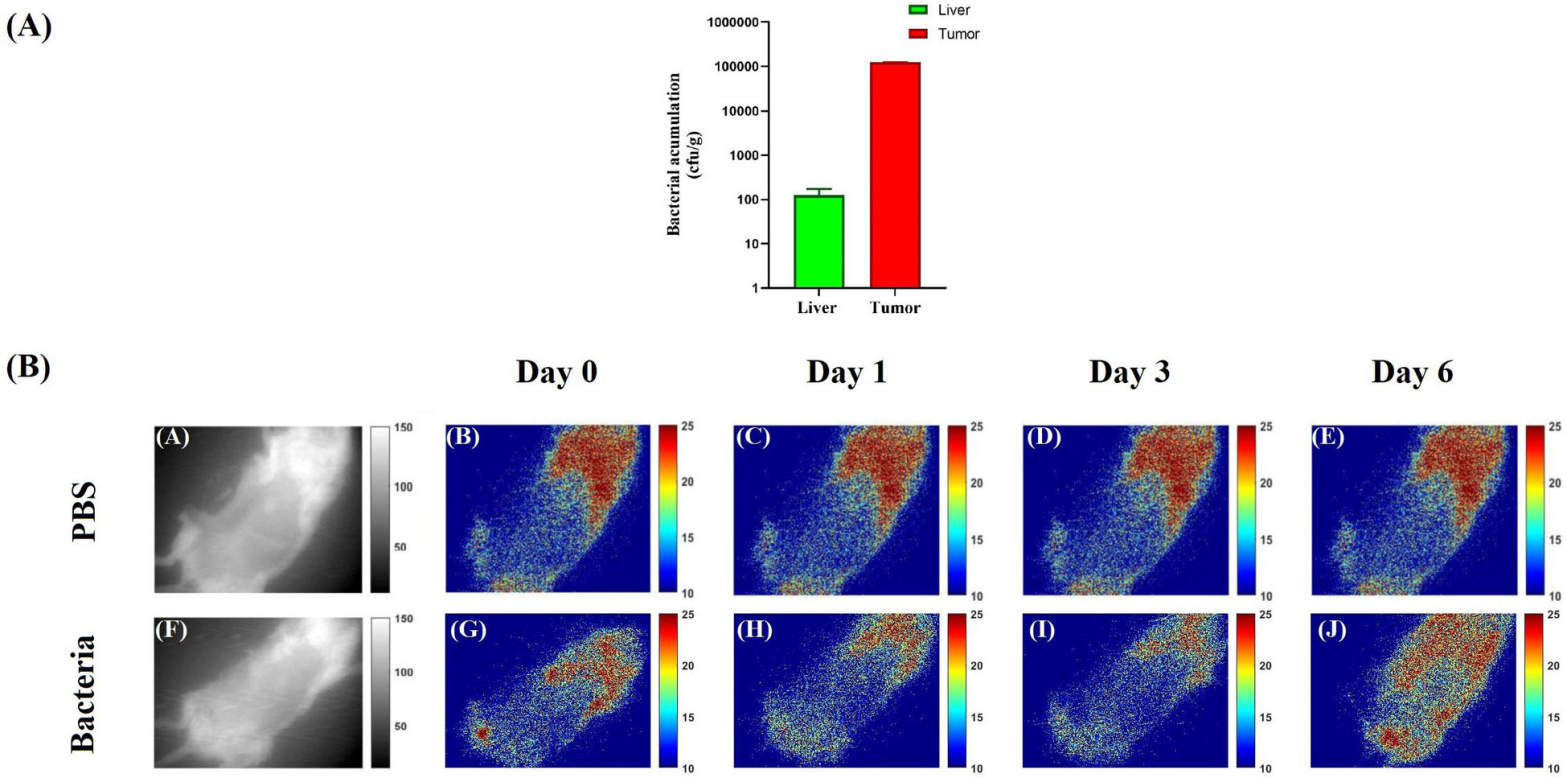
**A** Bacterial concentration in tumor tissue and liver of mice 48 h after IV administration of 108 cfu/g bacteria. Accumulation in tumor site was about 1000 folds greater compared to the liver. **B** Results of In vivo fluorescence reflectance imaging 0, 1, 3 and 6 days post injection. on day 6 post injection, Maximum New fluorescent emissions were completely restricted to the tumor site and reached to its maximum on day 6 post injection

Systemic administration of bacteria in syngeneic mice bearing spontaneous mammary tumors resulted in elicit and preferential colonization of bacteria at tumor site. As depicted in Fig. 4A, bacteria accumulated in tumor site with an approximately 1000 folds greater density compare to the liver. Furthermore, microscopic examination of tissue slides also confirmed that *E. coli* was present in all analyzed tumors. Accumulation of *E. coli BW25113* was more significant at necrotic sites of tumor.

### Confirming hypoxia-activated expression of cardiac peptides following tumor specific accumulation of *E. coli Bw25113* using in vivo fluorescence reflectance imaging

*E. coli BW25113* bearing cardiac peptides and GFP expressing construct and PBS were injected to the mice and images were collected immediately after and on days 1, 3 and 6 post injection. As depicted in Fig. 4B, no significant differences in emitted fluorescent intensity were recordable between two group during the first 24 h of the injection. On day 3 however, emitted fluorescent from engineered bacteria injected group was slightly higher than that of PBS receiving group. On day 6 post injection, emitted fluorescent from bacteria receiving group reached to its maximum amount and was completely restricted to the tumor site. Importantly, Fluorescent intensity of other organelles remained almost the same between control and treatment groups during image acquisition period. These observations further confirmed site specific colonization of bacteria and expression of GFP as an indicator of successful expression of construct in hypoxic condition.

### Tumor growth suppression potency of bacteria expressing cardiac protein

**Fig. 5:**
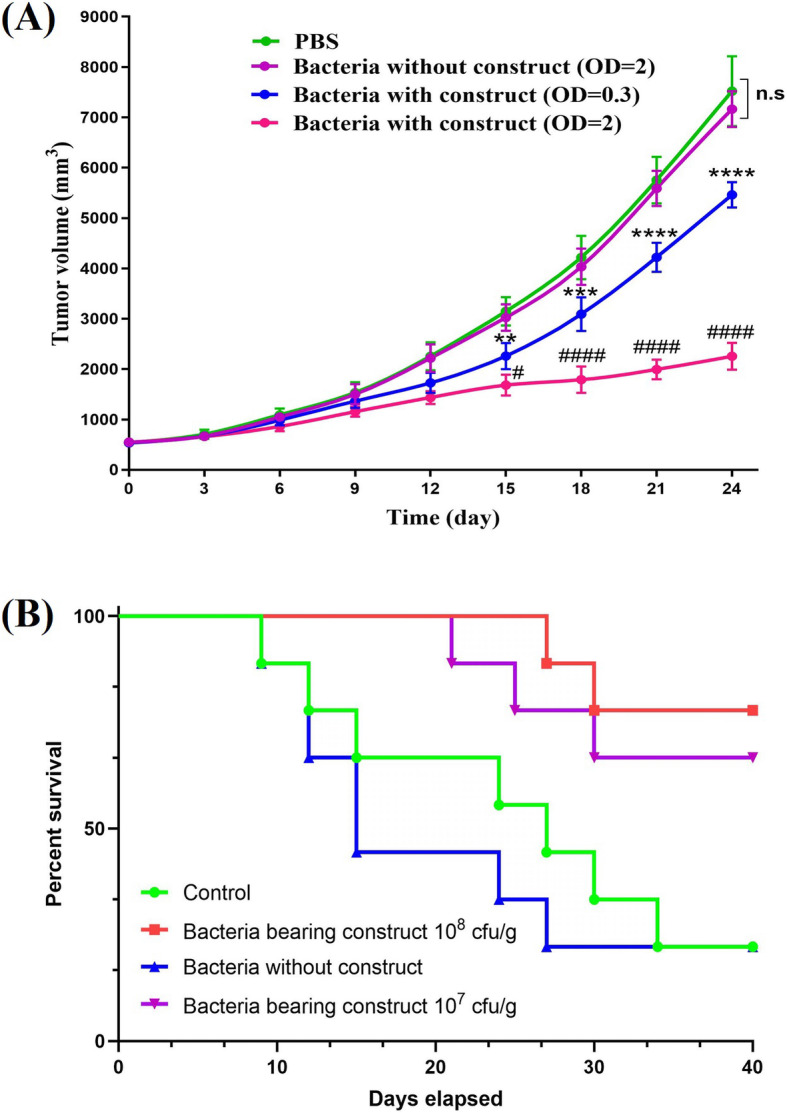
(**A**) suppression of tumor growth by administration of different concentrations of bacteria bearing construct. Increase in concentration of administered bacteria significantly enhanced tumor suppression potency. Ns: not significant, ****: *P* < 0.0001 compared to PBS, ####: P < 0.0001 compared to bacteria bearing construct at concentrations equal to 107 cfu/g (**B**) Results of Kaplan-Meyer analysis demonstrating a higher rate of survival in mice receiving bacteria bearing construct compared to PBS and bacteria without construct

Delay in tumor growth was evaluated over a 24-day period following administration of bacteria bearing construct at concentrations of 10^7^ and 10^8^ cfu/g, bacteria without construct at concentration of 10^8^ cfu/g and PBS as the control group. Based on results, the rate of tumor growth in mice receiving bacteria without cardiac peptides expressing construct and PBS was almost the same. Therefore, we concluded that *E. coli* bacteria per se were not capable of suppressing tumor growth speed. Contrarily, administration of bacteria bearing constructs, even at concentration as low as 10^7^ cfu/g could significantly suppress tumor growth rate in comparison to the PBS receiving group. Furthermore, increasing the concentration of bacteria from 10^7^ cfu/g to 10^8^ cfu/g resulted in a more intensified tumor suppression effect in vivo. As depicted in Fig. 5A, differences in extend of tumor growth suppression between two concentrations of injected bacteria bearing cardiac peptide expressing construct become significant on day 15 following the first injection. Moreover, differences between tumor growth rates of 10^8^ cfu/g engineered bacteria receiving group and PBS receiving group become significant from day 12 to the end of the study period. Finally, differences between tumor growth rates of 10^7^ cfu/g engineered bacteria receiving group and PBS receiving group become significant from day 15 to the end of the study period. Based on these observations we concluded that first, the delay in tumor growth observed with administration of engineered bacteria bearing cardiac expressing construct is mainly due to the secretion of cardiac proteins. Second, increasing concentration of administered bacteria may result in enhancement of production and secretion of cardiac peptides. Administration of bacteria bearing constructs at both concentrations also significantly enhanced survival rate as observed in Kaplan-Meyer analysis (Fig. 5B).

### Alteration in pro-inflammatory cytokine profile

**Fig. 6:**
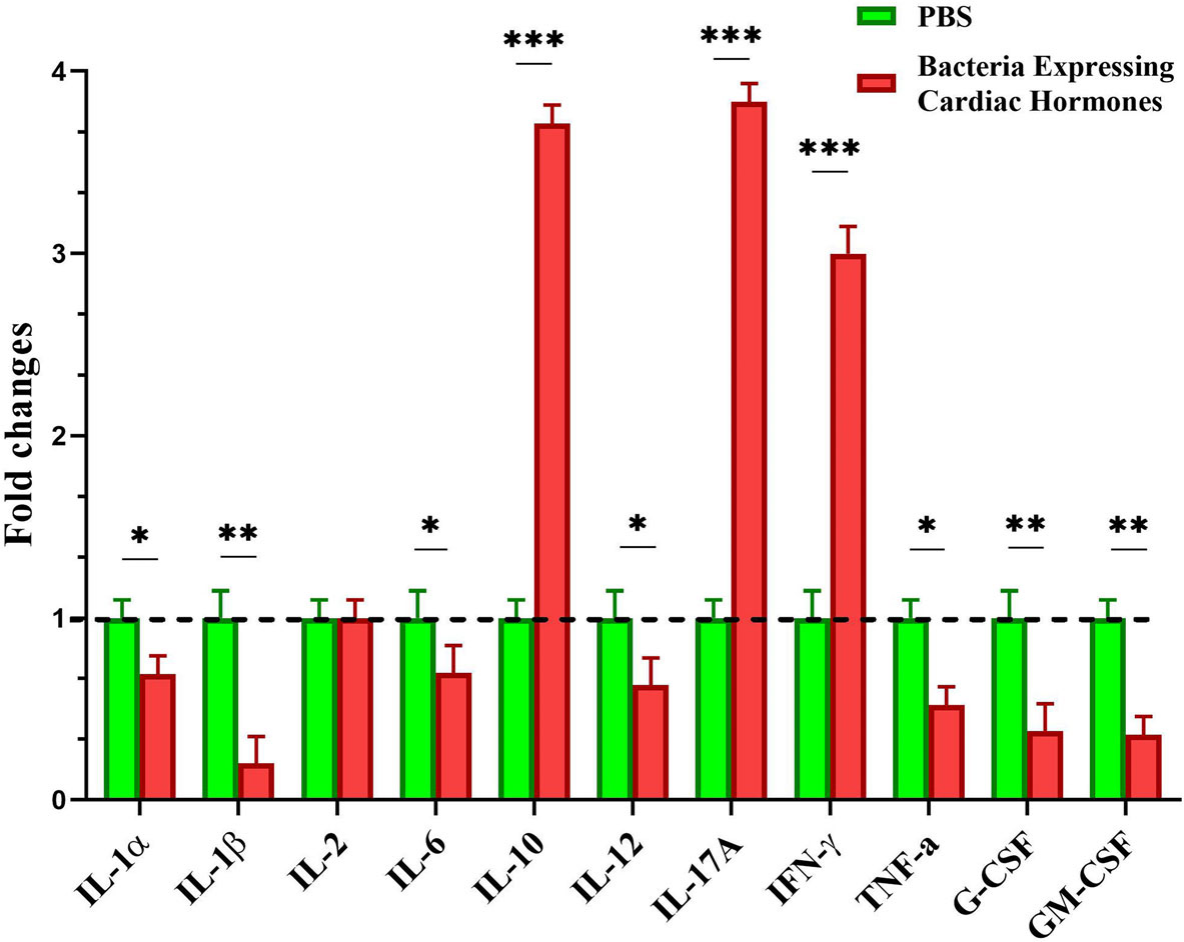
Alteration in expression of pro-inflammatory cytokines in response to bacterial therapy. Results are expressed as fold changes compared to control group receiving PBS. *: *P* < 0.05, **: *P* < 0.01, ***: *P* < 0.001 compared to control group

We also examined the effect of bacteria on expression of pro-inflammatory cytokines. As depicted in Fig. 6, administration of bacteria bearing construct at concentration of 10^8^ cfu/g body weight of mice with spontaneous breast tumors, resulted in a significant decline in expression of pro-inflammatory cytokines, including IL-1α, IL-1β, IL-6, IL-12, TNF-α, G-CSF and GM-CSF while increasing those of IL-17A, IL-10 and INF-γ compared to the PBS receiving group. Exceptionally, expression level of IL-2 pro-inflammatory cytokine was almost similar between two groups at the end of treatment period. Therefore, treatment with bacteria bearing cardiac hormone expressing construct could increase expression of some of the most important cytokines of both T_h_1 and T_h_2 cells (INF-γ and IL-10 respectively).

### Immunohistochemistry, quantification of micro-vessel density and H&E staining results

**Fig. 7:**
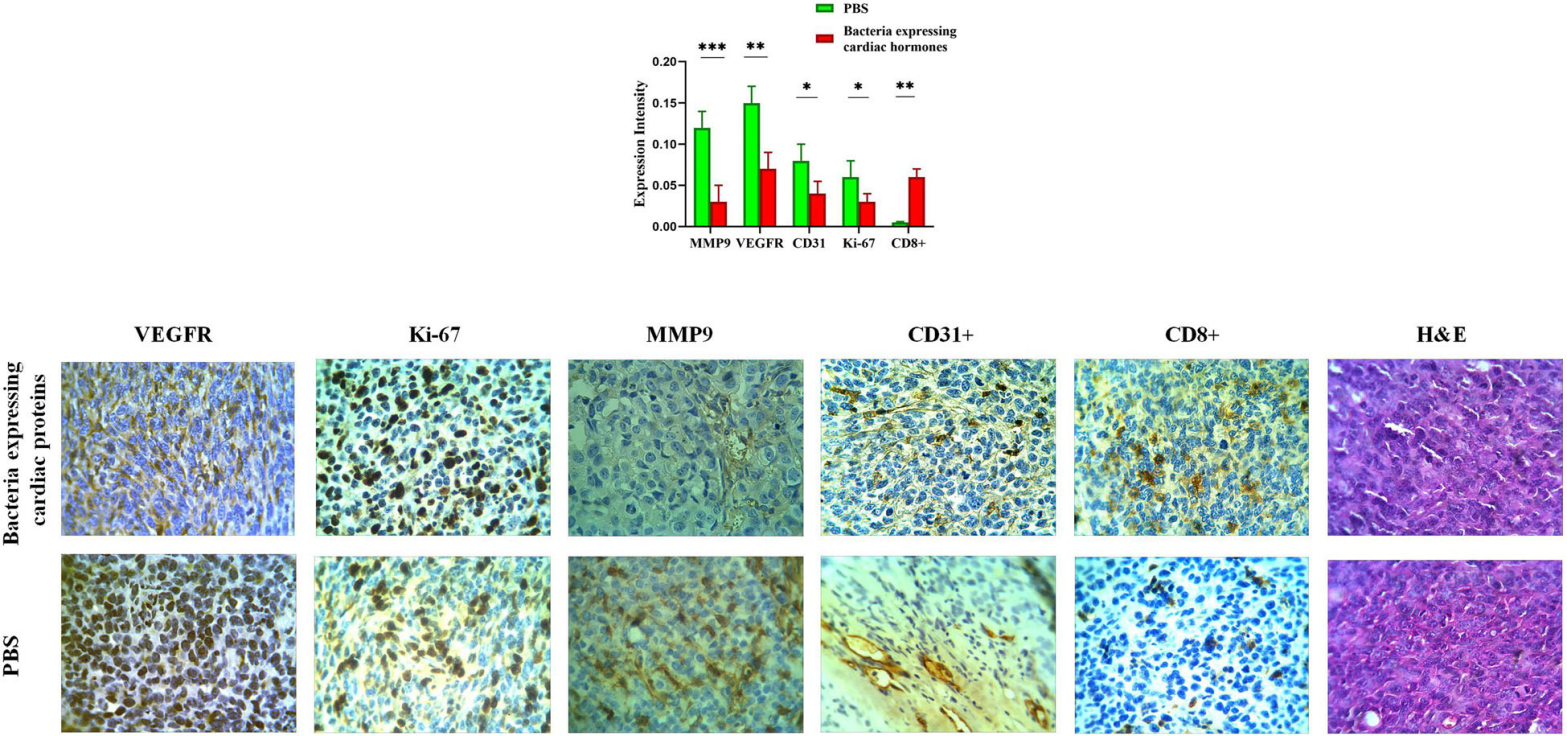
Results of immunohistochemical analysis, quantification of micro-vessel density and H&E staining. MMP9 expression was significantly decreased in mice receiving bacteria bearing construct at concentrations equal to 108 cfu/g compared to the PBS receiving group. Similarly, expression of VEGFR2 and Ki-67 was also significantly declined at the end of treatment period. Also, quantification of micro-vessel density by the CD31 marker in hot spots of sections demonstrated a significant decline in mice receiving 108 cfu bacteria bearing construct per gram of the body weight compare to the control group. Finally, the number of TILs and CD8+ TILs were significantly increased in mice treated with bacteria bearing construct at concentration equal to 108 cfu/g compared to the control group

As illustrated in Fig. 7, MMP9 expression was significantly decreased in mice receiving bacteria bearing construct at concentrations equal to 10^8^ cfu/g compared to the PBS receiving group. Similarly, expression of VEGFR2 and Ki-67 was also significantly declined at the end of treatment period. Also, quantification of micro-vessel density by the CD31 marker in hot spots of sections demonstrated a significant decline in mice receiving 10^8^ cfu bacteria bearing construct per gram of the body weight compare to the control group. Finally, the number of TILs and CD8+ TILs were significantly increased in mice treated with bacteria bearing construct at concentration equal to 10^8^ cfu/g compared to the control group.

## Discussion

In present study, we reported successful development of an engineered Hypoxia-responding *E. coli* BW25113 strain carrying cardiac peptides (LANP, VD and KP) and GFP (as an indicator protein) genes which could spatiotemporally target breast tumors, that is to say, preferentially delivering therapeutic cargo to the tumor site (spatial targeting) and expressing it only after receiving the stimulatory signal at tumor site (temporal targeting). Based on Stritzker et al., different strains of *E. coli* are capable of preferentially colonizing at tumor microenvironment [32]. Systemically administered bacteria to the blood stream are immediately distributed throughout the body and accumulate in both healthy and malignant tissues in almost equal numbers. Nevertheless, while residing bacteria in normal tissues and blood circulation are immediately excreted during first hours to days post injection, tumor accumulated bacteria proceed further in proliferation to reach a colony forming unit greatly exceeding that of the initially administered one. This Selective process of bacterial colonization in tumor microenvironment has been in large part attributed to the immunosuppressive nature of the tumor’s niche, resulted from pathological alterations induced in solid tumors [12].

Despite the existing evidence on antitumoral activities of bacteria, their localization and proliferation alone in tumor microenvironment is not sufficient for complete attenuation of the tumor growth. Hence, engineering bacteria to express potent cytostatic or cytotoxic proteins upon proliferation is of great importance in achieving an optimal therapeutic outcome. In this context, Kim et al., have shown that the growth of *E. coli* strain K-12 (MG1655) per se is not enough for attenuation of tumor growth in vivo [33]. Our results also clearly demonstrated that administration of *E. coli* strain BW25113, even at high doses, was not effective in reducing the rate of tumor growth. In contrast, bacteria bearing cardiac peptides construct could significantly attenuate the rate of tumor growth in vivo. Since the capability of expressing cardiac peptides is the only variance between transformed and non-transformed bacteria, differences in tumor growth rates may be attributed to the cardiac peptides, expressed under hypoxic condition of the tumor niche.

Secreted cardiac peptides are not so effective in promoting apoptosis but can effectively promote cell death in breast cancer cell lines after a 48-h incubation period in vitro and could suppress tumor growth and increase survival rate of tumor bearing mice in vivo. Consistently, Vesely et al. have reported that while the 4 cardiac peptides are capable of effectively inhibiting synthesis of DNA, their antitumoral effects are independent of apoptotic programmed cell death induction [34]. Moreover, several studies on lung, prostate and kidney cell lines have shown selective toxicity of cardiac peptides on cancer cells [35]. Pharmacological anticancer activities of LANP, VSDL and KP peptides have shown to begin upon interacting with natriuretic peptide receptor-A (NPR-A) and formation of cyclic GMP (cGMP) as a consequence of enhanced guanylate cyclase enzyme activity. Increased intracellular cGMP accumulation attenuates kinase activity in Ras/MEK/ERK cascade, ending in tumor growth suppression [24, 25]. Reported by Sun et al., VD and KP are capable of inhibiting RAS oncoproteins activities in the prostate cancer [36]. Mutated in up to 30% of the human malignancies, RAS proteins are the main members of the superfamily of small GTP binding and hydrolyzing proteins (GTPases) which are mainly responsible for transducing extracellular proliferative signals modulated by receptor tyrosine kinases (RTKs) in response to different groups of growth factors, including epidermal growth factor. Furthermore, LANP is capable of inhibiting 80–90% of the MEK1/2 and ERK1/2 activities in the prostate cancer cells [37, 38].

We also found that expression of VEGFR2 was significantly reduced on the surface of tumor cells in mice receiving bacteria bearing cardiac peptides expressing construct [26]. Angiogenesis is a firmly controlled process, crucial for tumor expansion beyond 2 to 3 mm in diameter and a critical step in tumor invasion and metastasis [39–41]. In parallel to their multi-kinase inhibitory activity, cardiac peptides are also capable of attenuating secretion of VEGFA, downregulate expression of VEGF receptor 2 (VEGFR2) expression, and inhibit activation of the Signal Transducer and Activator of Transcription 3 (STAT 3), all of which are effective approaches for suppressing pro-angiogenic responses in tumor site. Additionally, localization of ANP to the nuclear site of human pancreatic adenocarcinoma cell lines is indicative of possible interaction of these peptides with growth-promoting hormones there [42].

Alongside, at the molecular level, treatment with cardiac peptides has shown to induce a meaningful reduction in expression of β-catenin. Overall, in cancer cells, Wnt ligands bind with members of the Frizzled (Fz) family of serpentine receptors, as well as the LRP5 or LRP6 coreceptors to activate and recruit the phosphoprotein Dishevelled (DSH). Activated DSH in turn stabilizes the complex by involving axin and promotes the Akt-mediated inactivation of GSK-3β through phosphorylation of its serine amino acid at postion 9 [36]. This further results in stabilization and accumulation of β-catenin which upon translocation to the nucleus and binding with DNA modulates cellular proliferation rate, survival and most importantly, promotes epithelial to mesenchymal transition (EMT) of cancer cells, ending in promotion of cellular motility and metastasis [43]. Consistently, inhibiting continuous activation of Wnt/β-catenin signaling pathway observed in multiple studies, is a possible explanation of broad anticancer proliferation and metastasis activity of the cardiac peptides. Signaling cascades downstream of the activated β-catenin consists of c-JUN N-terminal kinase 2 (JNK2), VEGF and its corresponding receptor VEGFR2 [44]. Thus, application of cardiac peptides results in attenuation of Ras/MEK 1/2/ ERK 1/2 kinase cascade through restriction of Ras activation which can also suppress production of β-catenin itself and bring an end to the vicious cycle induced by aberrant activation of β-Catenin, VEGFR2 and activated Ras.

Another finding of present study was that administration of bacteria with cardiac peptides expressing constructs could reduce MMP-9 expression under hypoxic condition in vivo. Different studies have now suggested a key role for MMP9 in development of a proper microenvironment for promotion of tumor growth and angiogenesis by enhancing the interrelation between VEGF and VEGFR2 [45, 46]. This mainly takes place through the proteolytic cleavage and release of extracellular matrix (ECM) bound VEGF by MMP9 activity which is an important step in angiogenic switch. Based on Bergers et al. inhibitors of MMP9 effectively suppressed tumor growth and reduced angiogenic switching [47]. It has also been shown that MMP-cleaved VEGF plays an important role in tumors vascular patterning [26, 48]. Based on previous reports, cardiac peptides can effectively reduce expression of VEGFA in vitro [26]. In parallel, VEGFR2-triggered angiogenesis is considered as a hallmark of cancer progression and metastasis [49, 50].

Currently, multiple studies have addressed the role of cytokines in cancer proliferation, invasion and metastasis [51–55]. For instance, it has been shown that high circulating levels of IL-1β and IL-6 are unfavorable prognostic indicators and directly associate with higher risk of recurrence and more aggressive type of disease in patients with breast cancer [56–58]. Contrarily, overexpression of INF-γ has shown to be associated with increased rate of cell death through up-regulation of caspases and inhibition of angiogenesis [59–61]. Administration of bacteria bearing construct resulted in a complex effect on expression of pro-inflammatory cytokines. The anti-inflammatory behavior of cardiac peptides has been confirmed in multiple studies so far. For instance, it has been shown that ANP could down regulate expression of TNF-α which has an important role in alloreactivity of T-cells [62, 63]. Furthermore, ANP can also enhance expression of IL-10 which in turn, can suppress expression of IL-2 and proliferation of T-cells [64, 65]. Moreover, plasma concentration of IL-6 was also declined in response to the administration of ANP [65]. Finally, ANP treatment could decrease production of IL-12 by dendritic cells and generate fewer INF-γ positive T cells and much more IL-4 positive ones [66]. Consistently, a significant decline in peripheral blood concentrations of pro-inflammatory cytokines including IL-1a, IL-1b, IL-6, IL-12 and TNF-a was observed in present study while those of IL-10 and INF-γ were significantly increased in response to the treatment.

Results from different reports have shown that higher micro-vessel density values in tumor sites, measured by immunohistochemical analysis of CD31 expression, is correlated with higher rate of lymph node metastasis and associates with a poor prognosis outcome [67–69]. In parallel, MMP-9 enzyme is considered to be an important driver of cancer’s malignant progression, invasion and metastasis [70]. Determination of the proportion of proliferative cancer cells by Ki-67 immunostaining, is another effective marker for evaluation of the risk of metastasis and determination of the overall survival. In this context, greater Ki-67 positivity in tumor sections is together with a more invasive behavior of cancer cells and a poor overall survival in patients [71].

Alongside, different studies have confirmed anti-metastatic activity of cardiac peptides in vivo. Based on Nojiri et al., rate of metastasis in ANP treated mice following lipopolysaccharide treatment (mimicking surgical stress) was meaningfully reduced, suggesting metastasis preventive effects for these peptides. Consistently, the same group demonstrated that animals with endothelial cells, overexpressing ANP receptors possess a reduced rate of metastasis, which is in line with the previously mentioned fact that cardiac peptides could reduce the expression of VEGFA and downregulate the number of VEGFR2 receptors. More importantly, in addition to preventing formation of new metastatic lesions, cardiac peptide treatments can also help in dramatically reducing or even eliminating existing lesions [72]. Additionally, based on the fact that administration of bacteria bearing cardiac peptide construct in mice was together with an effective reduction in expression of Ki-67, CD31 and MMP-9 biomarkers, this method can be potentially considered as an effective approach for preventing cancer cells invasion and development of future metastases.

Finally, presence of TILs in high number inside tumor microenvironment (especially CD8+ TILs representing effective anti-tumoral activity), is considered to be a favorable prognosis value in different neoplasms [73–76]. Therefore, a significant increase in the survival rate of mice bearing tumors, revealed by Kaplan-Meyer analysis, may also be partly attributed to the higher number of CD8+ TILs in tumor site.

While the polycistronic design of the construct guarantees co-transcription of all inserted genes and observation of both high molecular weight GFP and low molecular weight cardiac peptide bonds in SDS-PAGE gels, confirms correct co-expression of all genes, lack of a confirmatory MALD-TOF/TOF data for verification of the correct expression of each peptide and determination of their concentration ratio in purified supernatant is one of the limitations of the present study. Nevertheless, mentioning this point is necessary that the purpose of the present study was to establish a spatiotemporal targeted bacterial based tumor delivery system with a strong anti-neoplastic activity.

Overall, in present study, we demonstrated that IV administrated *E. coli BW25113* strain bearing cardiac peptide’s expressing construct could specifically accumulate in tumor microenvironment and subsequently begin to secret cardiac peptides under the hypoxic nature of the niche. This resulted in suppression of tumor growth, a significant increase in survival rate of mice bearing breast tumors and a meaningful decline in expression of MMP-9, VEGFR2, Ki-67 and CD31 biomarkers, suggestive of antiangiogenic and metastatic properties for bacteria encompassing cardiac peptides expression cassette.

## Data Availability

Not applicable.
