# Correction: Engineered hypoxia-responding *Escherichia coli* carrying cardiac peptide genes, suppresses tumor growth, angiogenesis and metastasis in vivo

**DOI:** 10.1186/s13036-022-00297-6

**Published:** 2022-07-19

**Authors:** Mitra Samadi, Keivan Majidzadeh-A, Malihe Salehi, Neda Jalili, Zeinab Noorinejad, Marjan Mosayebzadeh, Ahad Muhammadnejad, Azadeh Sharif Khatibi, Shima Moradi-Kalbolandi, Leila Farahmand

**Affiliations:** 1 Recombinant Proteins Department, Breast Cancer Research Center, Motamed Cancer Institute, ACECR, Tehran, Iran; 2 Cancer Biology Research Center, Cancer Institute of Iran, Tehran University of Medical Sciences, Tehran, Iran

**Correction: J Biol Eng 15, 20 (2021)**

**https://doi.org/10.1186/s13036-021-00269-2**

Following publication of the original article [1], the authors reported missing information in the ‘Funding’ section.

The statement in the ‘Funding’ section originally read: Not applicable.

The statement in the ‘Funding’ section should read: Financial support for this project was kindly provided by the Iran National Science foundation (INSF).

The original article [1] has been updated.
